# Some Fever Hospitals and Their Work

**Published:** 1912-07-13

**Authors:** A. Knyvett Gordon

**Affiliations:** formerly Medical Superintendent of Monsall Hospital and Lecturer on Infectious Diseases in the University of Manchester.


### SOME FEVER HOSPITALS AND THEIR WORK.

### I.—Cross-Infection in Special Wards.

By A. KNYVETT GORDON, M.B. Cantab., formerly Medical Superintendent of Monsall Hospital and Lecturer on Infectious Diseases in the University of Manchester.

The treatment of patients of the poorer class in isolation hospitals is, on the whole, a benefit to the community, and even more so to the great majority of the sufferers themselves; but the system has some disadvantages which, though varying widely in extent in different institutions, are yet sometimes productive of disaster to individual patients, and often cause the general public—quite illogically—to condemn isolation hospitals altogether, an attitude which by the by is occasionally discernible in some members of our own profession, who really ought to know better.

One of these evils is the risk to the patient of "cross-infection" or, in other words, of superadded disease, and I think we may profitably consider the factors on which the existence of this evil depends, and how they may be minimised in practice.

Really there are two kinds of cross-infection: a patient may contract diphtheria or measles for instance—an altogether different disease from the scarlet fever for which he was admitted—or he may enter with a mild attack of scarlet fever only, and may subsequently develop exacerbations of the same complaint which we know, or have strong reason to believe, have been derived by infection from other patients in the same ward. We have to recognise that some scarlatinal patients can infect others ostensibly suffering from the same disease.

Many years ago a ward in a certain isolation hospital contained only patients who had, or were convalescing from, mild attacks of scarlet fever, until a case of the septic or "anginose" type was admitted: four days later the patient in the next bed developed an intense sore-throat with subsequent sloughing of the tonsils, and ultimately seven others in the ward suffered from septic throats.

Inasmuch as no column for this occurrence could be found in the statistical sheets provided for the institution, it did not cause very much disturbance in the minds of the authorities, who were quite happy when they were informed that diphtheria bacilli had not been found in any of the cases!

Now the administration of a fever hospital is a series of compromises. The ideal plan would be to treat each patient as if he were in a private house—that is to say, in a ward by himself, with a separate nurse and set of appliances for each case. This, however, is manifestly impossible, but we have to remember that *each departure we are constrained to make from the ideal carries with it some risk* to the patients of cross-infection. We can compromise, however, much more safely on certain lines than on others.

For convenience we may divide the methods of infection in scarlet fever into four routes, though there is, from the scientific point of view, no real difference between them. Infection may be conveyed from one patient to another (*a*) by aërial convection; (*b*) by direct contact; (*c*) by direct transmission; (*d*) by indirect transmission.

*Aërial Convection.*—In this case it is supposed that when one patient in a ward contracts scarlet fever or one of its transmissible complications from another, the infection passes straight through the air from patient to patient. Formerly this was considered to be the most common method, and on this theory it used to be thought that all we had to do to prevent a highly infectious case contaminating others was to remove it to a separation ward, that is to say, a separate compartment or room (in the same block), and that we might then safely nurse him with the same staff. In practice, however, this is always utterly useless by itself—for a reason which will be referred to later—and we do not nowadays attach much importance to aërial convection in scarlet fever. Incidentally, were it to exist to any great extent, we should expect that the risk of infection would diminish with the distance, and that the patient in the next bed but one, say, would be more liable to infection than one at the end of the ward; but, in practice, this is not the case, and we seldom find that distance from the source of infection constitutes a safeguard.

*Direct Contact.*—This no doubt is a very possible method of infection, but it scarcely requires mention, as it should nowadays never be permitted to exist.

*Direct Transmission.*—This perhaps requires some explanation. By it we mean the conveying of infective matter by the hands, clothing, etc., of the attendants or by surgical instruments, or domestic appliances such as cups, plates, bed-pans, and so on, direct from one patient to another. Personally I believe this to be the most important factor, and it will be at once evident if this is correct that we must pay more attention to the training of the staff than to the construction of the wards. I do not, of course, wish for one moment to belittle the attempt made by architects and others to ensure free ventilation and abundance of air-space and isolation accommodation—these are all vital—but it too often happens that authorities spend money freely and even lavishly on these, and subsequently economise foolishly and even dangerously in the numbers and personnel of the staff. Unfortunately we are too often unwilling to spend money on what is not obvious to the eye. We do not believe what we cannot see.

*Indirect Transmission.*—In this case the infective matter is conveyed from one patient—by the breath or secretions, for instance—to some article in the ward with which another patient subsequently comes in contact. The difference between this and the former route is one of degree rather than of kind, but it is convenient to make a distinction from the administrative point of view, in that direct transmission is a conscious, and indirect conveyance an unconscious, act. The best example of the latter is to be found in dust infection. Here infective particles are breathed into the air of the ward, and when cold settle down as dust. If this is either not taken away at all, or—what is worse—is stirred up by the ordinary process of sweeping, it may settle on something which another patient may touch, perhaps a week later, and thus infect him.

Let us now see what machinery we have for dealing with the various ways in which infection may take place. Firstly, we have the important question of diagnosis, and here we see the paramount necessity for the existence of a resident medical officer. Where the hospital is administered, for instance, by a non-resident medical officer of health it is practically impossible for every patient to be seen on arrival, and in consequence diagnosis is left to the matron or head nurse! The first essential is, of course, to keep any patient who may infect others out of the ward if possible, so that we do not have to deal with the problems presented by an outbreak of measles, for instance, due to faulty diagnosis on admission.

Assuming, however, that we are correct in our diagnosis, and that all the patients in the ward are suffering from scarlet fever in one form or another, we have three ways of dealing with anyone whom we may consider to be dangerous to others. We can transfer him to a separation ward in the same block and nursed by the same staff; to an isolation ward—that is to say, to another building nursed by a different staff; or we can keep him in the ward and treat him by the "barrier" system.

The essential features of all barrier systems are:

(*a*) That the bed should either be merely labelled with a distinguishing mark, such as a red cord, or be surrounded with a circle of screens, which again may or may not be covered with wet antiseptic sheets.

(*b*) That the nurse should wear an overall whenever she attends to the patient in any way.

(*c*) That her hands should be protected (as regards other patients in the ward) either by the wearing of rubber gloves whenever she has to touch the patient or his utensils or clothing, or by washing them in disinfectant before she leaves the barrier.

(*d*) That all utensils for the "barriered" patient should be marked and kept separate, and washed up separately from those of the others in the ward.

The great advantage of the barrier is its elasticity; it does not depend upon the construction of the ward, and it can be improvised at once for any patient for whom it may be required; within the limits of the available staff as many barriers as may be required can be prescribed in any given ward.

The system ensures almost complete protection against infection by direct and indirect transmission, and—if screens be employed—practical protection against aërial convection also.

It is obvious, however, that the efficiency of the barrier system depends upon the intelligence and conscientiousness of the nursing staff, but that these can be secured by careful and intelligent training. My own experience is that nurses take a very keen interest in the details of the barrier system when they have once realised that the process is simply that of surgical asepsis. It is essential, however, that the staff in any ward should not be so overworked that it is physically impossible for them to attend to the details of the barrier. Unfortunately, it is often much more difficult to obtain nurses than buildings.

We come now to the application in practice of these safeguards at our disposal. We may divide the "dangerous" cases into three classes as follow:—

Those found on admission to be suffering from some other infectious disease in addition to scarlet fever, the commonest of these being measles, chicken-pox, whooping-cough, and diphtheria. If isolation wards are available it is best to utilise them for this type of case, because if spreading of the concomitant infection occurs it does comparatively little damage. Personally, I prefer to "barrier" these cases in addition to isolating them, unless one happens to have a ward containing some other patients with the same association of diseases.

(*b*) Those who, while suffering undoubtedly from scarlet fever, have been exposed before admission to other infections, which they may, or may not, therefore subsequently develop. For these the separation ward is the best, but it is as a rule quite safe to barrier them in the general ward until the maximum incubation period of the second disease has passed.

(*c*) The cases of septic scarlet fever, or scarlatina anginosa. For these, *which are undoubtedly dangerous to others*, the barrier is the method par excellence, and those who have had experience of the results obtained when septic cases were ignored as far as any nursing precautions went, and of wards of the same type with barriers in working order, cannot fail to be greatly impressed with the improvement under the latter system.

Besides those that are dangerous to others, there are patients to whom an ordinary scarlet fever ward is itself harmful—namely, those in whom the diagnosis of scarlet fever is doubtful, and I need hardly say that it is almost a crime to admit a patient to a general ward without precautions unless one is quite certain from one's own observation that he really is suffering from scarlet fever, however suspicious the history may have been. For these the best place is an isolation block which is reserved for cases of this type—that is to say, which does not receive cases of coexistent infection also. If this is not available, the barrier in the general ward is an excellent substitute, and it should be continued until it is certain that the patient has, or has not, scarlet fever. But it does not do to admit to a general ward, even with a barrier, patients who turn out to be really suffering from measles, so, if there is any history of exposure to this infection, it is best to use the isolation ward *ab initio*. Measles, incidentally, is the bugbear of the resident medical officer, inasmuch as it is the only disease that is so infectious as to require the separation of the staff in attendance on it from their fellows, even when they are off duty altogether; nurses, for instance, in a measles ward should not be allowed to enter the common sitting-rooms.

